# Editorial: Disease Ecology and Biogeography

**DOI:** 10.3389/fvets.2021.765825

**Published:** 2021-10-29

**Authors:** Luis E. Escobar, Serge Morand

**Affiliations:** ^1^Department of Fish and Wildlife Conservation, Virginia Tech, Blacksburg, VA, United States; ^2^Global Change Center, Virginia Tech, Blacksburg, VA, United States; ^3^Center for Emerging Zoonotic and Arthropod-Borne Pathogens, Virginia Tech, Blacksburg, VA, United States; ^4^Doctorado en Agrociencias, Facultad de Ciencias Agropecuarias, Universidad de La Salle, Bogotá, Colombia; ^5^CNRS ISEM—CIRAD ASTRE, Montpellier University, Montpellier, France; ^6^Faculty of Veterinary Technology, Kasetsart University, Bangkok, Thailand; ^7^Faculty of Tropical Medicine, Mahidol University, Bangkok, Thailand

**Keywords:** *Anaplasma*, *Borrelia*, *Caligus*, dengue, disease ecology, disease biogeography, *Toxoplasma*, vector-borne

## Introduction

Disease ecology and disease biogeography are complementary fields for the study of infectious diseases, both fields focus on the distribution and abundance of pathogens across scales. While disease ecology focuses on the study of disease systems at the local level and during short periods, disease biogeography expands the study of disease systems over large areas and prolonged periods. What is local vs. regional extents and what is short vs. prolonged periods is debatable. We propose that the study of the ecology and biogeography of disease systems takes place along a continuum, were the limits of ecology and biogeography are blurred. To demonstrate that disease systems operate from the local to the global and can be tracked from the fine to the coarse, this Research Topic compiles study cases that highlight concepts, methods, and applications of ecology and biogeography to untangle spatial patterns of disease phenomena across scales and for diverse infectious diseases.

## Infectious Diseases Across Scales

### Local

Niehaus et al. provide a local-level characterization of the ecology of *Toxoplasma gondii* in wildlife across diverse landscape configurations. *Toxoplasma gondii* is a generalist protozoan parasite affecting hundreds of mammals and birds, and is particularly virulent in some species of Neotropical primates. In their study, Niehaus et al. employed a modified agglutination test to detect antibodies against *T. gondii* in serum samples from captive and free-ranging Neotropical primates from Costa Rica, a small country in Central America. The authors found widespread exposure of howler (*Alouatta palliata*), spider (*Ateles geoffroyi*), capuchin (*Cebus imitator*), and squirrel (*Saimiri oerstedii*) monkeys to *T. gondii*. Captive primates revealed higher seroprevalence (59.6%) compared to free-ranging wild primates (11.6%). Nevertheless, among free-ranging animals seroprevalence varied among species, which could be relevant for some primates of conservation concern and for which *T. gondii* infections are particularly virulent. Niehaus et al. report that landscape factors such as percentage of forest cover and precipitation could explain *T. gondii* exposure in primates. This finding suggests that local-level microclimatic conditions could play a role in the maintenance of *T. gondii* in the environment, facilitating infections in wildlife.

Winter et al. (1) provide a local-level assessment of a prion disease in wildlife to determine the extent to which infection risk can be predicted by the landscape configuration. In their study, Winter et al. examined Chronic Wasting Disease in white-tailed deer (*Odocoileus virginianus*) in the north portion of Virginia, United States. Chronic Wasting Disease is caused by a pathogenic prion protein for which there is limited information about its ecology and biogeography. To address the question “Is the distribution of Chronic Wasting Disease predictable by landscape data?” the authors used a machine learning protocol to contrast the distribution of cases of positive deer against satellite-derived vegetation data during the 2009–2020 period. After an exhaustive evaluation of the predictive capabilities of the models, the authors concluded that Chronic Wasting Disease has a measurable and predictable ecological signal expressed as the capacities of models to predict the distribution of new cases better than by chance based solely on satellite-derived vegetation data. The mechanisms behind this phenomenon remain unknown but could be related to (i) models mirroring the ecology of deer or (ii) models reconstructing landscapes where prion transmission is more likely. The last hypothesis is supported by the fact that Chronic Wasting Disease prions remain infective during long periods (years) in the environment and that specific types of soil and vegetation have proved to augment the infectivity of prions (2). This opens the door for more exciting assessment of the role of the landscape on the epidemiology of infectious prions in wildlife.

Although fish parasites are impressively diverse and have received comprehensive attention from evolutionary and ecological approaches (3), the literature on fish disease ecology and biogeography is scarce at least. To help fill this gap of knowledge, Lepe-Lopez et al. developed a latitudinal assessment of the ecology and geography of the sea louse (*Caligus rogercresseyi*), which is among the most relevant parasite affecting salmonid fishes. Authors explored the distributional range of sea louse along the coastal areas of southern Chile, a medium-size country in South America. In their study, Lepe-Lopez et al. linked sea louse locality reports with data of sea water temperature, salinity, and current velocity to reconstruct the suitable areas for the species to disperse and, in turn, infect salmonids. For a more comprehensive assessment of the biogeography of sea louse, Lepe-Lopez et al. projected their models to the next decades and across diverse climate change scenarios. Authors were able to reconstruct the distributional expansion, contraction, and shift of sea louse for the next 80 years across a large latitudinal gradient, which has implications to the native fishes and the intensive aquaculture industry in Chile. This contribution provides detailed recommendations to the control and management of sea louse in salmon in Chile and also brings important insights on the biogeography of parasites of marine fishes.

### Regional

At a larger geographic extent, Gettings et al. assessed the spatial and temporal trends of *Anaplasma* spp. and *Borrelia burgdorferi*, two widespread zoonotic pathogens, across the continental United States. The study included over 30 million serological test results for *Anaplasma* spp. and *B. burgdorferi* in dogs during the 2013 and 2019 period. Using a Bayesian spatio-temporal binomial regression model, the authors found spatial correlation on the distribution of cases: higher prevalences clustered in neighbor counties, and prevalence decreased when geographic distance increased. Gettings et al. studies show that *Anaplasma* spp. and *B. burgdorferi* occur in tractable and predictable “hotspots” where transmission is more likely, especially along the Northeast region of the United States. The authors note a plausible relationship between the rise of *Anaplasma* spp. seroprevalence and cases of human anaplasmosis. This study exemplifies the role of dogs as sentinels of zoonotic pathogens transmitted by ticks at the continental scale. Similarly, detection of patterns of decrease in prevalence of these pathogens in dogs in some endemic areas could represent encouraging effects of public education and use of preventative practices. Overall, the study concludes in that the data suggests an increase in seroprevalence of *Anaplasma* spp. and *B. burgdorferi* in areas known to already have high prevalence.

### Continental

Robles-Fernández et al. explored aspects of the biogeography of dengue spillover using a macroecological approach. The authors measured the linkages between species known to be susceptible to dengue virus infection and those unknown to be susceptible. Linkages were measured based on geographic, climatic, and phylogenetic similarities between a set of 10 wildlife species broadly reported susceptible to dengue and a set of 1,778 species of mammals across the America. The authors measured the geographic, climatic, and phylogenetic distances between known and unknown dengue hosts to determine the likely host breath of dengue virus across the continent. Their analytical approach combines datasets from diverse sources, at different scales, and from hundreds of taxa, integrated using correlative machine learning classification tools. Their multiscale analytical framework was guided using biogeographic theory that overcomes previous methods to predict suitable hosts for pathogens based solely on phylogenetic distances and traits of candidate hosts. From this disease biogeography approach, we learned that dengue transmission can occur in specific areas, across narrow landscape characteristics, and in predictable wildlife species. More specifically, Robles-Fernández et al. found that wildlife susceptibility to dengue infection was more likely among species phylogenetically close to known hosts, occurring in warm and arid landscapes, and distributed in the tropics. Among the potential new hosts of dengue, the common vampire bat (*Desmodus rotundus*) was predicted with the highest probability.

### Global

Morand and Lajaunie assessed the potential relationships between human infectious diseases and deforestation and expansion of monoculture crops. In their study, the authors measured data on forest cover, oil-palm monoculture harvested area, and human demography during the period 1990 and 2016, combined with outbreak data from 116 zoonotic and 69 vector-borne diseases for the same period. The study revealed an alarming rate of increase in the number of zoonotic and vector-borne diseases outbreak in the last three decades. Morand and Lajaunie also found that, during this period, the area of oil palm monoculture has dramatically increased at the same rate of disease outbreaks. Indeed, the study reports significant association between the increase of oil palm and vector-borne diseases, especially in tropical countries. Nevertheless, relationships between landscape change and disease were asynchronous among global regions. For instance, tropical regions showed high forest loss in association with increasing outbreaks of vector-borne diseases, while temperate regions showed forest gain associated with increasing outbreaks. Together, Morand and Lajaunie studies add to the limited available quantitative evidence linking deforestation with human infectious diseases.

## From Local to Global, From Ecology to Biogeography

The local-level studies of *Toxoplasma gondii* antibodies in primates, prion-derived Chronic Wasting Disease in deer, and sea louse in salmonids were conducted at spatial resolutions going from hundreds of meters to a few kilometers. Thus, these investigations elucidate elements acting locally on the ecology of the disease systems studied. The regional-level study of zoonotic vector-borne pathogens in dogs used counties as the finest geographic unit and moths as the finest temporal unit to cover a comprehensive portion of the North America region. The study of likely wildlife hosts of dengue virus in the Americas used data form the molecular level, to the climatic level at ~20 km, to the geographic contexts covering entire species ranges that included multiple Latin American countries. A global-level study explored the effects of global landscape change on the risk of zoonotic diseases and revealed that patterns found in tropical regions are not necessarily consistent with patterns in many temperate countries. This overview of disease ecology and biogeography exemplifies the need for multiscalar assessments of infectious diseases, going from ecology to assess local processes, to biogeography to assess continental and global patterns.

Local-level disease investigations were the most frequent study cases presented in this Research Topic. Local-level disease ecology research has advanced our understanding of the spread of infectious diseases. Abundant local-level studies and limited availability of regional- and global-level studies seem to be the dominant pattern in disease ecology research (4). Thus, more research is needed at a coarse scale to fill gaps of knowledge on disease biogeography.

This Research Topic highlights five important challenges for the future of disease ecology and disease biogeography. First, drivers of disease transmission found important at one scale are not necessarily consistent at a different scale. Second, most studies use proxies of disease, including data of previous exposure to the pathogen (e.g., serology), data of the presence of the source of transmission (e.g., disease vector), or data of the source of the pathogen (e.g., disease reservoir); thus, ecology and biogeography research accounting for the pathogen *per se* is needed. Third, due in large part to data limitations, models are simplified views of complex host-pathogen dynamics. That is, most disease modeling experiments assume species to be susceptible or resistant to infection (a binary representation of infection), instead of considering susceptibility as a continuous dimension where different levels of susceptibility are considered across individuals, populations, and species. Fourth, empirical evidence suggests that the capacities of a pathogen to cause disease, as a decrease in the fitness of the host, also known as virulence, is also a property of the host, not only of the pathogen (3). In other words, a pathogen can be virulent to one host species but not to others. Changes in pathogen virulence among host taxa, however, is rarely considered in disease ecology and biogeography. Fifth, disease is assumed by the solely presence of a microorganism or the presence of antibodies. As a result, many models may overestimate the potential burden and risk of infectious diseases. Finally, this Research Topic gives and overview of the state of the art regarding disease ecology and biogeography, and provides examples of big-data applications, artificial intelligence approximations, multiscale multidimensional assessments, and multisectoral collaboration. We see a promising future of disease ecology and biogeography with practical applications to conservation medicine, veterinary epidemiology, and public health, and encourage active collaboration among veterinarians, ecologists, and biogeographers to generate exciting and innovative studies on the distribution and abundance of pathogens, their vectors, and reservoirs, and the host populations at risk of infection.

## Author Contributions

LEE and SM were involved in writing the article and agreed to the final version of this editorial. All authors contributed to the article and approved the submitted version.

## Funding

LEE was supported by the Center for Emerging, Zoonotic, and Arthropod-borne Pathogens (CeZAP) Interdisciplinary Team-building Pilot Grant, the Virginia Department of Wildlife Resources, and the National Science Foundation Human-Environment and Geographical Sciences Program, grant 2116748. SM is supported by French ANR project FutureHealthSEA (ANR-17-CE35-0003-01).

## Conflict of Interest

The authors declare that the research was conducted in the absence of any commercial or financial relationships that could be construed as a potential conflict of interest.

## Publisher's Note

All claims expressed in this article are solely those of the authors and do not necessarily represent those of their affiliated organizations, or those of the publisher, the editors and the reviewers. Any product that may be evaluated in this article, or claim that may be made by its manufacturer, is not guaranteed or endorsed by the publisher.

## References

[1] Winter Steven N Kirchgessner Megan S Frimpong Emmanuel A Escobar Luis E. A landscape epidemiological approach for predicting Chronic Wasting Disease: a case study in Virginia, US. Front Vet Sci. (2021) 8:698767. 10.3389/fvets.2021.698767 34504887 PMC8421794

[2] Escobar LE Pritzkow S Winter SN Grear DA Kirchgessner MS Dominguez-Villegas E. The ecology of chronic wasting disease in wildlife. Biol Rev. (2020) 95:393–408. 10.1111/brv.12568 31750623 PMC7085120

[3] Morand S Krasnov BR Littlewood DTJ. Parasite Diversity and Diversification. Cambridge: Cambridge University Press (2015).

[4] Winter SN Escobar LE. Chronic wasting disease modeling: an overview. J Wildl Dis. (2020) 56:741–58. 10.7589/2019-08-213 32544029

